# Effects of Feeding Fermented Mulberry Leaf Powder on Growth Performance, Slaughter Performance, and Meat Quality in Chicken Broilers

**DOI:** 10.3390/ani11113294

**Published:** 2021-11-18

**Authors:** Yanan Ding, Xiaodie Jiang, Xiaofeng Yao, Haihan Zhang, Zehe Song, Xi He, Rong Cao

**Affiliations:** 1 College of Animal Science and Technology, Hunan Agricultural University, Changsha 410128, China; dingyanan13@163.com (Y.D.); jxd_0120@163.com (X.J.); yaoxiaofeng0525@163.com (X.Y.); zhhous@163.com (H.Z.); zehesong111@163.com (Z.S.); 2 Hunan Engineering Research Center of Poultry Production Safety, Changsha 410128, China; 3 Ministry of Education Engineering Research Center of Feed Safety and Efficient Use, Changsha 410128, China

**Keywords:** fermented mulberry leaf powder, broiler chicken, meat quality, growth, slaughter performance

## Abstract

**Simple Summary:**

Mulberry leaf is widely used in ruminants feeding, such as sheep, beef cattle, and dairy calves. Due to the high content of crude fiber in mature mulberry leaves and branches and the presence of anti-nutritional factors such as tannin, excessive addition will affect the production performance and health of livestock and poultry, and limit its large-scale application in animal production to a certain extent. The disadvantages of woody plants can be improved by microbial fermentation, which can reduce the content of anti-nutritional factors, and increase the content of peptides and amino acids, probiotics, and bioactive components. In this study, *Lactobacillus*, *Saccharomycetes*, and *Bacillus subtilis* were used to make mixed strains to ferment mulberry leaf powder, and different proportions were added to the diet of yellow feathered chicken broilers. The results showed that the addition of fermented mulberry leaf in the diet could improve the digestion and absorption of nutrients, and then improve its growth performance, and increase the contents of inosine monophosphate (IMP), total amino acids, essential amino acids, and delicious amino acids in breast and thigh muscle, and improved polyunsaturated fatty acids and essential fatty acids in breast muscle; this also has a positive effect on improving meat quality.

**Abstract:**

This study was conducted to investigate the effects of feeding fermented mulberry leaf powder (FMLP) on growth performance, slaughter performance, and meat quality of broilers. A total of 360 1-day-old chickens were randomly divided into 5 groups. The control group was fed basal diet (CON), 3% FMLP, 6% FMLP, 9% FMLP, and 3% unfermented mulberry leaf powder. The (MLP) group was fed basal diet supplemented with 3%, 6%, 9% fermented mulberry leaf powder, and 3% MLP, respectively. The experiment lasted for 56 days, with 1–28 days as the starter phase and 29–56 days as the grower phase. The results on the growth performance showed that diets supplemented with 3% FMLP significantly increased the ratio of villus height to crypt depth in the duodenum, jejunum, and ileum of broilers, enhanced the activity of intestinal amylase and digestibility of dry matter and crude protein, improved the average daily gain (ADG), and decreased the feed to gain ratio (F/G) (*p* < 0.05). Compared with the control group diet, the 3% FMLP group diet significantly increased the breast muscle yield (*p* < 0.05), reduced the abdominal fat ratio (0.1 < *p* < 0.05), and improved the slaughter performance of broilers. The 3% MLP group diet increased the shear force of breast muscle (*p* < 0.05) and thigh muscle of broilers compared to the control group, and adding FMLP could reverse the above results. Additionally, relative to the control group, FMLP supplementation improved the contents of inosine monophosphate (IMP), total amino acids (TAA), essential amino acids (EAA), and delicious amino acids (DAA) in breast and thigh muscle, and improved polyunsaturated fatty acids (PUFA) and essential fatty acids (EFA) in breast muscle; the 6% and 9% FMLP groups showed preferably such effects (*p* < 0.05). In conclusion, dietary supplementation of FMLP can improve the digestion and absorption of nutrients, and then improve the growth performance of broilers; it also has a positive effect on improving slaughter performance and meat quality.

## 1. Introduction

With the development of population and the improvement of people’s living standards, the demand for livestock and poultry production and the conventional feed resources is increasing. The shortage of feedstuff has become increasingly prominent and the price of conventional feedstuff with large consumption has gradually risen. Therefore, finding cheap and reasonable feed resources to replace conventional feedstuff has become a research hotspot and strategic future after the COVID-19 outbreak [1].

Mulberry is a deciduous perennial woody plant, belonging to Morus of Moraceae. Its leaves are considered as a high-quality forage plant resource because of its rich crude protein content (22~29.8%), balanced amino acid composition, rich in vitamins, trace elements, phytosterols, flavonoids, alkaloids, polysaccharides and other bioactive substances [2,3], and so on. However, due to the high content of crude fiber in mulberry leaves and branches and the presence of anti-nutritional factors such as tannin, the excessive addition of mulberry leaves and branches would affect the production performance and health of livestock and poultry, which, to a certain extent, limits its large-scale use in animal production [4]. The related disadvantages of woody plants could be improved by a microbial fermentation treatment, which reduced the content of anti-nutritional factors, increased the content of polypeptides and amino acids, and contains a variety of beneficial products such as probiotics bioactive ingredients [5]. Studies have found that adding fermented mulberry leaves into feeds could enhance immunity [6], regulated lipid metabolism [7], and improved the quality of animal products [8]. In conclusion, fermented mulberry leaf, as a new protein feed resource, has a broad application prospect in animal husbandry production.

Mulberry leaves as unconventional feed resources are mainly used in ruminants, such as sheep [9], beef cattle [10], and dairy calves [11]. Fermented feed can be used to improve the intestinal health of broilers [12,13] and growth performance [14,15] has been reported. However, there are few studies on the application of mulberry leaves in poultry production after fermentation.

In recent years, probiotic fermentation technology has become a powerful tool to reduce anti-nutritional factors in feed, and improve nutritional quality and the bioavailability of nutrients [16,17]. Therefore, in this study, the mixed strains of *Lactobacillus*, *Saccharomycetes*, and *Bacillus subtilis* were used to ferment mulberry leaf powder to investigate the effects of fermented mulberry leaf powder on production performance, slaughter performance, and meat quality of broilers, so as to provide a theoretical basis for the application of fermented mulberry leaf in livestock production, especially in areas where mulberry leaves are widely planted.

## 2. Materials and Methods

### 2.1. Preparation of FMLP Sample

Mulberry leaf powder (MLP) and Fermented mulberry leaf powder (FMLP), which were made from the leaves of hybrid feed mulberry, also known as Yajin protein mulberry, were provided by Hunan Institute of Sericulture Science. Fermentation strains (*Lactobacillus*, *Saccharomycetes* and *Bacillus subtilis* = 1:2:1, viable count ≥ 3 × 10^9^ cfu/g), provided by Shandong Kangdien Biotechnology Co., Ltd. (Linyi, China). FMLP was prepared by solid-state fermentation for one week. After laboratory testing, the routine nutrients of MLP and FMLP were obtained and are shown in Table 1.

### 2.2. Experimental Birds and Feeding

All of the experimental procedures were approved by the Animal Care and Use Committee of Hunan Agricultural University. In total, 360 one-day-old male yellow-feathered broilers provided by Hunan Xiangjia Animal Husbandry Co., Ltd. (Hunan, China) were randomly divided into five groups, consisting of 6 replicates of 12 birds each, which was then denoted as CON group (basal diet), 3% MLP group (basal diet supplemented with 3% mulberry leaf powder), 3%, 6%, and 9% FMLP group (basal diet supplemented with 3%, 6%, and 9% fermented mulberry leaf powder). The addition dosage of FMLP was adjusted accordingly, on the basis of the study of Has et al. [18]. All birds were raised in wire cages with 3-level battery following standard temperature regimens, which gradually decreased from 32 to 25 °C. The lighting scheme was all day lighting, throughout the test. Meanwhile, birds were offered basal diet and diet supplemented with mulberry leaf powder and different doses of fermented mulberry leaf powder and provided ad libitum access to water and diet in crumbled (1–28 d) and pelleted form (29–56 d). The experiment lasted for 56 days. The basal diets of the starter (1–28 d) and grower phase (29–56 d) formulated according to the feeding standard of chicken (NY/T 33-2004) are shown in Table 2.

### 2.3. Sample Collection

At 56 d of age, after 8 h of starvation, 6 birds (1 bird per replicate) were randomly selected from each treatment group. The weight of broilers after plucking and bloodletting was taken as dressed weight (DW) and after removal of head, foot, and viscera was taken as eviscerated weight (EW). Dressing percentages was calculated by DW/BW. Eviscerated yield was calculated as the percentages of BW. Breast muscle, thigh muscle, and abdominal fat pad including leaf fat surrounding the cloaca and gizzard were separated and weighed. Breast and thigh muscle yields were calculated as the percentages of EW. Abdominal fat percentage was calculated by abdominal fat weight/(abdominal fat weight + EW). Subsequently, within 10 min postmortem, all the right entire pectoralis majors and thigh muscle were collected for the determination of meat quality. Parts of the pectoralis major and thigh muscle samples were cut from the same location, quickly frozen in liquid nitrogen, and then kept at −80 °C for further analysis.

### 2.4. Growth Performance

Feed intake was recorded weekly, and total feed consumption in each replicate were recorded at 1, 28, and 56 d to determine average daily feed intake (ADFI), average daily gain (ADG), and feed to gain ratio (F/G).

### 2.5. Apparent Nutrient Digestibility

During the experiment, 0.5% titanium dioxide (TiO2) was added to the diet as an exogenous indicator. The basic diet and four experimental diets were fed to the different treatment groups respectively. The first 3 days were used to adapt the birds while in the last 3 days, about 300 g of representative fecal samples were selected from each replicate every day, pooled, weighed, oven-dried (55 °C), milled, and stored pending chemical analyses. Apparent digestibility values for crude fibre, crude protein were calculated according to the following formula:

AD (%) = 100 − [(G1 × F2)/(G2 × F1)] × 100

AD: apparent digestibility of dietary nutrients, G1: titanium content in diet, F1: nutrient content in the diet, G2: titanium content in feces, F2: nutrient content in feces.

### 2.6. Intestinal Digestive Enzyme Activity

After slaughtering, the intestines of the experimental chickens were taken out, and the middle part of the jejunum about 10 cm was separated with a scalpel. The contents of the jejunum were put into a centrifuge tube, frozen in liquid nitrogen and stored at −80 °C. Amylase (Amy), lipase (LIP), and protease (PT) in jejunum contents were determined by commercial kits (Nanjing Jiancheng Bioengineering Institute, Nanjing, China), according to the manufacturer’s recommendations.

### 2.7. Intestinal Histomorphology

Briefly, the intestinal samples were dehydrated with increasing concentrations of ethanol, cleared with xylene (Surgipath Medical Industries, Richmond, IL, USA), and embedded with paraffin wax (Thermo fisher scientific, Kalamazoo, MC, USA), and cut into 4-μm thick histological sections for hematoxylin and eosin staining. The tissue sections were measured under a microscope using a 40 × combined magnification, and an image processing and analysis system (Version 1, Leica Imaging Systems Ltd., Cambridge, UK). Villus height (VH); villus width (VW); crypt depth (CD); and VH/CD ratio (VH:CD) of the small intestine were determined by Program Image-pro Plus 6.0.

### 2.8. Meat Quality

The meat color was measured at 60 min postmortem from a mean of three random readings made with a portable chromameter (CR-300, Minolta, Japan), which was calibrated with a white tile according to the manufacturer’s manual. At 45 min and 24 h after slaughtering, the pH of breast and thigh muscles were measured with a pH meter (pH-STAR, SFK technology, Denmark), previously calibrated with pH 4.6 and 7.0 buffers. The drip loss of breast and thigh meat was determined as described by Zhang et al. [19]. In brief, take a 3 × 2 × 1 cm piece from position of each sample of breast and thigh meat to determine drip loss. This sample was weighed and the mass was recorded as W1, and then suspended from a hook and placed in an inflatable zip–lock bag with the direction of the muscle fiber parallel to the gravity direction and hung for 24 h at 4 °C. After 24 h, the sample was removed and cleaned of moisture using filter paper, then weighed to obtain W2. Drip loss was then calculated as a percentage, where drip loss (%) = (W1 − W2)/W1 × 100%. L* (lightness), a* (redness), and b* (yellowness) of five random locations surface of the chicken breast and thigh meat were measured using a colorimeter (Konica Minolta Sensing Inc., Osaka, Japan) 1 h postmortem [20]. Cooked breast and thigh meat were cooled to room temperature and then rectangular-shaped samples (1 × 1 × 2 cm) at the same location were removed to measure tenderness using a TA-XT2 texture analyzer (Stable Micro Systems, Godalming, UK) with a Warner-Bratzler blade (code HDP/BS, Stable Micro Systems). Shear force was measured perpendicular to the axis of muscle fibers in 6 replicates for each treatment.

### 2.9. Muscle Chemical Analysis

About 50 g breast and thigh muscles samples were sliced up, weighed, placed in a weighing bottle, and reweighed. The weighing bottle was placed into a freeze dryer at 50 °C for 48 h, and then reweighed. The weight difference between the initial sample and the dried sample was used to calculate the moisture percentage. Then, the dried samples were powdered with Muller CS-700 (Wuyi Haina Electric Appliance Co., Ltd., Zhejiang, China) and used for the analysis of crude protein (CP), amino acid and fatty acid composition. The crude protein, ether extract (EE), and crude fiber (CF) content were analyzed according to the method of the Association of Official Analytical Chemists.

### 2.10. Inosine Monophosphate Content Measurement

About 5 g fresh muscle samples were weighed into a 15 mL centrifuge tube and homogenized in ice bath at 10,000 rpm for 30 s with t-25 ultra turrax homogenizer (IKA, Staufen, Germany). Then, we weighed 2.5 g of homogenate into a 50 mL centrifuge tube and added 25 mL of 5% perchloric acid. After shaking, it was centrifuged at 3500 rpm for 10 min in 4 °C refrigerated centrifuge, and then filtered into a 100 mL beaker. Then, 15 mL of 5% perchloric acid was added into the centrifuge tube. This was shaken well for 5 min, then centrifuged again and the two filtrates were mixed. After adjusting the pH to 6.5 with 5 mol/L and 0.5 mol/L NaOH, the filtrate was transferred into a 100 mL volumetric flask and diluted to the calibration tail with ultrapure water. The samples were filtered into the automatic vial and then used for HPLC.

### 2.11. Amino Acid Composition of Muscle

About 150 mg dried breast and thigh muscle were weighed into a glass bottle and 15 mL of 6 mol HCl were added. After nitrogen filling, the mixture was hydrolyzed for 22–24 h at 110 °C. Next, the hydrolysate was transferred to a 50 mL volumetric flask and diluted to calibration tail with ultrapure water. The solution was filtered using a 0.45 μm membrane filter into an autosampler vial, and then analyzed by L-8900 amino acid analyzer (HITACHI, Japan).

### 2.12. Fatty Acid Composition of Muscle

Lipid extraction from breast muscle samples was performed by the Folch et al. method [17]. The extracted lipid was hydrolyzed in 2 mL KOH–methanol (C = 0.5 mol/L). After shaking for 1 min, the mixture was reacted in 95 °C water for 10 min to obtain a mixture of free fatty acids. The free fatty acid mixture was esterified in 2 mL BF3–methanol solution (W = 10%). After shaking for 10 s, the mixture was reacted in 80 °C water for 20 min. Subsequently, adding 1 mL n-hexane and 5 mL saturated NaCl solution, mixed for 1 min, then centrifuged for 15 min at 3000 rpm. Next, a volume of 800 μL fatty acid methyl esters was separated and analyzed with a GC-2010 plus gas chromatograph (Shimadzu, Japan). The injector and detector temperatures were maintained at 250 °C and 260 °C, respectively. Nitrogen was used as carrier gas, and the flow rate was 2.5 mL/min. The column temperature profile was as follows: maintained at 100 °C for 5 min, increased to 180 °C at 8 °C/min, increased to 210 °C at 4 °C/min, and maintained at 210 °C for 5 min. Next, the temperature was raised to 230 °C at 10 °C/min and then kept unchanged for 10 min. Fatty acids could be identified by comparing the retention time of the peaks with known standards (Sigma, St. Louis, MO, USA).

### 2.13. Statistical Analyses

Data are expressed as the mean ± standard deviation. Statistical analysis of the index was carried out according to the replicate of each group. The differences among the groups were analyzed by One-Way Analysis of Variance (ANOVA) followed by Tukey’s test using the SPSS 22.0 software (SPSS, Chicago, IL, USA). Significance was set at *p* < 0.05.

## 3. Results

### 3.1. Growth Performance

The effects of dietary supplementation of FMLP on growth performance are presented in Table 3. In the starter phase, ADG increased by 11.44%, 10.46% (*p* < 0.01); F/G decreased by 15.88%, 10.59% (*p* < 0.05), respectively, in chicken receiving 3% mulberry leaf powder and fermented mulberry leaf powder meal compared to those given basal diet group. Moreover, the ADFI in 3% fermented mulberry leaf powder group was significantly increased by 7.52% (*p* < 0.05) compared to the control group. In the grower phase, compared with the 3% mulberry leaf powder group, the ADG of the 3% fermented mulberry leaf powder group was significantly increased by 14.24% (*p* < 0.05); the ADFI and F/G of broilers among all groups had no significant differences (*p* > 0.05); adding low dose of fermented mulberry leaf powder had a trend to increase the ADFI of broilers (0.05 *< p <* 0.10). In the entire experimental phase, compared to the control group, the ADG of broilers in the 3% fermented mulberry leaf powder group was dramatically increased by 18.39% (*p* < 0.05), and the F/G of broilers in the 3% fermented mulberry leaf powder group was sharply decreased by 10.88% (*p* < 0.05).

### 3.2. Apparent Nutrient Digestibility

The dry matter (DM), CP digestibility of broilers in FMLP group were improved, and the 3% FMLP group were markedly increased by 6.98%, 10.36%, respectively (*p* < 0.05, Table 4) compared to the control group. Ether extract (EE) digestibility improved by 5.98%, 3.84%, and 9.89% (*p* < 0.05) in chicken receiving 3%, 6%, 9% FMLP meal compared to those given basal diet group. The digestibility of EE and ASH of broilers in all experimental groups were increased compared to the control group, but there was no significant difference between them (*p* > 0.05).

### 3.3. Intestinal Digestive Enzyme Activity

The digestive enzyme activity of amylase, lipase, protease in jejunum of broilers are shown in Table 5. Compared with the control group, the activities of amylase in jejunum of broilers in the 3%, 6%, 9% FMLP groups and 3% MLP group were significantly increased by 15.33%, 13.63%, 11.93%, and 19.46%, respectively (*p* < 0.01, Table 5). The lipase activity in jejunum of broilers in the 9% FMLP group was significantly increased by 24.44% (*p* < 0.05), and in other experimental groups it was increased, but there was no significant difference (*p* > 0.05) compared to the control group.

### 3.4. Intestinal Histomorphology

The normal function and structure of the intestinal tract were indicated by the villus height, crypt depth, and villus length/crypt depth (V/C), as shown in Table 6. The duodenal villus height of broilers in each experimental group was increased (*p* > 0.05), and the duodenal crypt depth was lower than that in the control group (*p* > 0.05). Compared with the control group, the duodenal V/C ratio of broilers in each dose of FMLP group was markedly increased by 25.69%, 19.71%, and 33.72% (*p* < 0.05). Compared to the control group, the villus height of jejunum in the 9% FMLP group was sharply decreased by 31.07% (*p* < 0.05), and the V/C ratio of jejunum in the 3% FMLP group was significantly increased (*p* < 0.05). The ileal crypt depth of broilers in each group decreased (*p* > 0.05), and the ileal V/C value of broilers in the 3% and 9% FMLP groups and in the 3% MLP group increased by 23.06%, 16.71%, and 18.27%, respectively (*p* < 0.05) compared to the control group.

### 3.5. Slaughter Performance

As shown in Table 7, the breast muscle yield of broilers in the 3% FMLP group was markedly increased by 8.38% (*p* < 0.05), compared with the control group. In addition, the abdominal fat percentage of broilers in the 3% MLP group was sharply decreased by 29.68% (*p* < 0.05), and the abdominal fat percentage of broilers in other treatment groups had a decreasing trend (0.1 < *p* < 0.05) compared to the control group. There were no significant differences in dressing percentage, eviscerated carcass yield, thigh muscle yield among the treatment groups (*p* > 0.05).

### 3.6. Meat Quality

The meat quality of the breast and thigh muscle fed with diets containing different doses of FMLP and MLP are summarized in Table 8. The L* value of thigh muscle was decreased (*p* > 0.05) and that of breast muscle was increased by adding FMLP to broiler diet, and in the 9% FMLP group, it was significantly increased by 16.45% (*p* < 0.05). Adding FMLP to broiler diet could reduce the a* value of thigh muscle (*p* > 0.05), but increased the a* value of breast muscle. Compared with the control group, the b* value of breast muscle in broiler diets supplemented with FMLP had no significant effect (*p* > 0.05), but it could reduce the b* value of thigh muscle, and in the 6% and 9% FMLP groups, it decreased by 27.80% and 25.65%, respectively (*p* < 0.05). Compared to the control group, the 3% MLP group increased in muscle shear force of breast muscle (*p* < 0.05) and thigh muscle (*p* > 0.05). However, the shear force of breast muscle and thigh muscle in the FMLP group decreased, especially in the 6% and 9% FMLP groups, in which it markedly (*p* < 0.05) decreased, compared with the 3% MLP group. Adding FMLP and MLP to the broiler diet could reduce the drip loss of breast and thigh muscles (*p* > 0.05). In addition, there were no significant effect on the ph_45min_ value and ph_24h_ value of broiler muscles (*p* > 0.05).

### 3.7. Muscle Chemical Composition and Inosine Monophosphate Content

The chemical composition including the moisture, EE, CP, and IMP content in breast and thigh muscle is presented in Table 9. The IMP of breast muscle increased (*p* < 0.05) by 31.11, 35.55, and 32.92%, the IMP of thigh muscle increased by 16.77, 19.88 (*p* < 0.05), and 24.84% (*p* < 0.05) in chickens fed 3, 6, and 9% FMLP diet compared to those given basal diet. There were no significant differences in the contents of moisture, EE, and CP in the muscle of broilers among the groups (*p* > 0.05).

### 3.8. Amino Acid Profile

Dietary supplementation of FMLP increased the contents of total TAA, EAA, and DAA in breast muscle and thigh muscle (Table 10). Concentrations of TAA in the 6% and 9% FMLP groups were significantly increased by 2.84% and 3.23%, and in the thigh muscle were remarkably increased by 4.16% and 4.39% compared with those in the control group, respectively (*p* < 0.05, Table 10). Compared with the control group, seven EAA (Try was not detected) in breast and thigh muscles of the diet treatment group were increased (Table 10 and Table S1). Lys and Val in breast muscles of the 3%, 6%, and 9% FMLP groups were significantly increased by 4.46%, 5.94%, 7.92% and 13.27%, and 14.16% and 15.04%, compared to the control group, respectively (*p* < 0.05, Table 10 and Table S1). Lys, Met, Phe, and Val in the 6% and 9% FMLP groups were significantly increased by 4.5% and 7.66%, 16.33% and 30.61%, 5.98% and 10.26%, an 11.38% and 13.82%, respectively (*p* < 0.05, Table 10 and Table S1). Compared to the control group, supplementing FMLP in the diet could significantly increase the content of DAA in the muscle of experimental chickens (*p* < 0.05, Table 10). Except Gly, Tyr, and Phe, the DAA concentrations in breast muscle of each diet treatment group were remarkably higher than those of the control group (*p* < 0.05, Table 10 and Table S1). Concentrations of DAA (except Val and Gly) in thigh muscles of the 6% and 9% FMLP groups were remarkably higher than those of the control group, in which Asp, Glu, Ala, and Phe were markedly increased by 6.78% and 9.60%, 3.00% and 11.97%, 16.52% and 25%, and 5.98% and 10.26%, respectively (*p* < 0.05, Table 10 and Table S1).

### 3.9. Fatty Acid Profile

The fatty acids detected in the breast and thigh muscles of broilers were basically the same, mainly composed of C16:0, C18:0, C18:1n-9t, and C18: 2n-6*, as shown in Table 11 and Table S2. Compared to the control group, the C18:1n-9t in the breast muscle of MLP group and each dose FMLP group increased 5.22%, 5.93%, 7.36%, and 12.54% (*p* < 0.05, Table S2). Concentrations of C22: 6n-3*, C18: 2n-6*, and C20: 4n-6* in the breast muscle of the control group were remarkably increased in each group (*p* < 0.01, Table S2), among which the C22: 6n-3* increased by 13.15%, 15.79%, 18.42%, and 23.68%, and C18: 2n-6* increased by 15.32%, 21.07%, 13.79%, and 24.77%, and C20: 4n-6* increased by 28.67%, 21.67%, 20.97%, and 23.08%, respectively. In addition, the concentrations of PUFA and EFA in the dietary treatment group was significantly higher than that in the control group (*p* < 0.05, Table 11), and the content of MUFA in the breast muscle had a tendency to increase (0.05 < *p* < 0.1, Table 11). Compared with the control group, the C16:1n-7 content in broiler thigh muscle was increased by adding FMLP in the diet, and the C16:1n-7 content in the 6% and 9% FMLP groups was significantly increased by 22.71% and 18.98% (*p* < 0.05, Table S2). Supplementation of 3%, 6%, and 9% FMLP significantly improved the content of C18:3n-6* in thigh muscle of broilers compared to the control group (*p* < 0.05, Table S2).

## 4. Discussion

Mulberry leaf, as a new type of feed resource, has a great potential for development and utilization in animal production due to its characteristics of large yield and balanced nutrition. However, its large-scale use is limited due to the fact that its mature leaves and stems contain anti-nutritional factors such as tannin. Fermentation, especially probiotic fermentation, has attracted more and more attention, because of its potential to reduce dietary anti-nutritional factors, improve feed nutritional quality, and promote animal growth performance [13,14]. Our results showed that, relative to the control group, dietary supplementation of low-dose FMLP markedly increased the ADG and sharply decreased the F/G in the starter phase, remarkably increased the ADFI, and significantly decreased the F/G in the whole experiment period, but the effect was weakened with the increase of the supplemental dose. Nutrient digestion and absorption may play an important role in improving growth performance. Previous studies have also found that the addition of 10% and 20% fermented and unfermented mulberry leaves in the diet of broilers significantly reduced the final body weight and dietary dry matter and crude protein digestibility of broilers with the increase of mulberry leaves supplemental dose [18]. This study showed that dietary supplemented with 3% FMLP could improve the digestibility of dry matter and crude protein nutrients of broilers, and the digestibility decreased with the increase of supplemental dose compared to the control group, which was consistent with the findings of Has et al. [18]. The digestibility decreased with the increase of supplemental dose, which may be attributed to the accelerated digestion rate caused by the increase of dietary fiber content, thus reducing the digestion time of nutrients and the digestion and absorption of nutrients by the gastrointestinal tract [21], and affecting the retention of nutrients (dry matter, organic matter, nitrogen) [22], because the fiber content of MLP and FMLP is higher, with 12.30% and 8.67%, respectively. In addition, in this study, relative to 3% MLP group, the F/G of broilers in the 3% FMLP group markedly decreased from 1 to 56 days. The reason for this result was related to the reduction of antinutritional factors in the diet and the degradation of macromolecular organic matter into small molecular by fermentation which is easy to be absorbed and utilized and the presence of probiotics in the diet, so as to improve the nutrient absorption and animal growth performance [23,24]. Other factors affecting nutrient absorption, such as digestive enzyme activity and intestinal morphology, were also examined. The results showed that dietary supplementation of FMLP can remarkably increase the activity of the intestinal amylase. Relevant studies have demonstrated that adding fermented feed and probiotics to broiler diet can improve the activity of intestinal digestive enzymes, which may be connected to the metabolism of probiotics in the intestine to produce part of digestive enzymes and improve the activity of related digestive enzymes [24,25]. Normal intestinal function and structure are the biological basis for growth and nutrient digestion and absorption of animals [26]. Villus height, crypt depth, and ratio of villus height to crypt depth (V/C) are important indexes to evaluate intestinal digestion and absorption in animals. The higher the villi height, the better the intestinal digestion and absorption function [27]. In this study, dietary supplementation MLP markedly increased ileum V/C value, and supplementation of FMLP significantly increased duodenum, jejunum, and ileum V/C value of broilers, with the 3% FMLP group having the most significant effect. These results were consistent with the study by Feng et al. [28], which revealed improved intestinal tissue morphological structure and increased intestinal digestive enzyme activities by adding fermented feed to broilers’ diets. According to the present results, it is suggested that dietary supplementation of FMLP promotes the growth performance of broilers by improving intestinal tissue structure, digestive enzyme activity, and nutrient digestibility.

Slaughter performance is an important index to measure the carcass quality of meat livestock and poultry. It can not only directly reflect the percentage of the mass of different tissue parts in the total mass, but also reflect the difference of the deposition amount of nutrients in different tissue parts. High abdominal fat in broiler chickens will directly affect the processing of meat products, reduce slaughter rate and consumers’ purchase desire, and affect economic benefits [8]. In this study, it was found that the supplementation of MLP and FMLP in the diet of broilers can reduce the abdominal fat percentage of broilers, which may be relevant to the regulation of active substances in mulberry leaves on fat metabolism of broilers. Previous studies have demonstrated that 1-deoxynojirimycin (DNJ), the main alkaloid in mulberry leaves, had the effect of lowering blood glucose and blood lipid [29]. In our study, diets supplementing with FMLP had no effect on dressing percentage, eviscerated carcass yield, and thigh muscle yield of broilers, which was consistent with the finding of Semjon et al. [30]. In addition, diets supplementing with 3% FMLP may significantly increase the breast muscle yield of broilers, which may be related to the improvement of the digestibility of crude protein.

Meat color is an important appearance index of meat quality, which directly affects consumers’ purchasing desire [31]. Indicators reflecting meat color are L*, a*, and b* [32]. Within a certain range, higher the a* value, the better the quality and freshness of the meat; the higher the L* value of meat color, the higher the gloss of the meat and the paler the color. The a* value is directly proportional to meat quality, while the b* and L* values are inversely proportional to meat quality [33]. Our results showed that the addition of 9% FMLP significantly reduced the b* of thigh muscle and increased the L* value of breast muscle, which indicated that the different types of muscle fibers might be the reason for the different effects of FMLP on different muscle tissues of broilers [34]. Probiotics and active substances may play an important role in the effect of dietary FMLP on meat color of broilers. This conjecture is consistent with the findings of Yu et al. [35], who reported that dietary supplementation of high concentrations had the most significant effect on meat color [35,36,37], and the findings of Shen et al. [38], who revealed that the effect of adding bamboo leaf extract in the diets with different concentrations on meat color was linearly increased [38]. Tenderness (shear force) may be the most important edible quality parameter that determines consumer acceptance [39]. Shear force is an intuitive indicator of muscle tenderness [40]. In the present study, the addition of FMLP could reduce muscle shear force, especially at medium and high doses. Probiotics in fermented mulberry leaf powder may play an important role in it. Previous studies similarly have found that dietary supplementation probiotic can reduce muscle shear force [35,41]. Relative to the control group, the muscle shear force of MLP group was significantly increased, which may be related to the increase of ADG, resulting in the increase of muscle fiber diameter, which in turn led to the increase of shear force, because the smaller the muscle fiber diameter is, the more tender the muscle is [42].

The composition and content of amino acids, fatty acids, and nucleotides flavor substance in animal and poultry meat are important factors that affect the nutritional value and flavor. In the present study, compared to the control group, diets supplementing with FMLP remarkably improved the content of IMP, increased the contents of EAA, TAA, and DAA, and the effect of medium and high dosage FMLP were most significant, but dietary supplementation MLP had little effect on the IMP content in breast and thigh muscle of broilers, suggesting that probiotics may play an important role in the FMLP group. Previous studies similarly have demonstrated that diet supplementing with 5% alfalfa (similar to MLP, it can be used as unconventional protein feed) meal did not affect IMP of breast and thigh muscles; dietary supplementation probiotics increased the content of IMP, DAA, EAA, and DAA in breast muscle [35]. In addition, in the present study, fatty acids in breast muscle and thigh muscle of broilers were mainly C16:0, C18:0, C18:1n-9t, and C18:2n-6. Their total contents accounted for a significant proportion in the total fatty acid composition, and they were the main component of muscle fatty acids in broilers, and unsaturated fatty acids were the main component, which was consistent with the findings of Semjon et al. [30]. Farmer et al. [43] illuminated PUFA are more likely to form volatile flavor substances during lipid oxidation, which makes meat more delicious. C18:2n-6, C18:3n-6, and C20:4n-6 are EFA indispensable to the human body in PUFA, which play a very important role in maintaining normal development and health, and can effectively prevent atherosclerosis and myocardial infarction [44]. C18:3n-6 and C22:6n-3 are important raw materials for the formation of biofilms, which promote the development of the nervous system and brain [45]. Our result showed that dietary supplementation of MLP and FMLP markedly increased the content of PUFA (C18:1n-9t, C18:2n-6, C20:4n-6, C22:6n-3) and EAA in breast muscle of broilers, indicating that active substances and probiotics may play an important role in MLP and FMLP groups. Previous studies similarly have reported that diets supplementing fermented ginkgo biloba leaves increased the contents of flavonoids and polysaccharides in diets, and then increased the content of total PUFA in breast muscles [46]; diets supplementing with probiotics improved the content of PUFA and SFA in breast muscles [47]. Additionally, dietary supplementation with MLP and FMLP had little effect on thigh muscle, only improved the content of C18:3n-6 in muscle, which indicated that the effects of MLP and FMLP on fatty acid content of muscle in different parts of broilers were different, which might be caused by potential differences in nutrient absorption and distribution among different tissues [48]. According to the present results, it is suggested that dietary supplementation with MLP and FMLP can improve the nutritional value and flavor of meat by affecting the composition and content of PUFA in muscle of broilers.

## 5. Conclusions

Diet supplementing with FMLP at a dosage of 3% could improve the digestion and absorption of nutrients, such as the digestibility of dry matter, CP and EE, amylase and V/C ratio, and then improve growth performance. Adding 6% and 9% FMLP could improve the meat quality of breast and thigh muscles without affecting the growth performance, such as increasing the concentration of IMP, TAA, EAA, DAA, PUFA, and EFA, reducing the shear force, and the 9% FMLP group showed preferably such effects.

## Figures and Tables

**Table 1:** Routine nutrient analysis of MLP and FMLP.

Item ^1^	CP (%) ^1^	EE (%) ^1^	CF (%) ^1^	Ash (%)	Ca (%) ^1^	TP (%) ^1^
MLP	16.9	3.00	12.3	12.4	2.73	0.420
FMLP	20.5	3.07	8.67	11.6	2.66	0.490

^1^ MLP: mulberry leaf powder; FMLP: fermented mulberry leaf powder; CP: crude protein; EE: ether extract; CF: crude fiber; Ca: calcium; TP: total phosphorus.

**Table 2:** Ingredients and nutrient compositions of experimental diets of starter and grower phase.

Item	Starter Phase (1–28 d)	Grower Phase (29–56 d)
Ingredients (%)		
Corn	64.0	66.3
Soybean meal	26.0	25.6
Wheat bran	0.600	1.00
Fish meal	2.00	0
Soybean oil	1.70	3.30
Calcium hydrogen phosphate	1.70	1.40
Limestone	1.28	1.20
Premix ^1^	2.72	2.80
Total	100	100
Nutrient levels ^2^		
ME (MJ/kg)	12.1	12.6
Crude protein	19.2	17.5
Calcium	0.920	0.730
Total phosphorus	0.410	0.860
Lysine	1.18	1.05
Methionine	0.470	0.450

^1^ Premix provided per kilogram of diet: Fe, 80 mg; Cu, 8 mg; Zn, 60 mg; Mn, 80 mg; I, 0.70 mg; Se, 0.3 mg; vitamin A, 6000 IU; vitamin D_3_, 1000 IU; vitamin E 20 IU; vitamin K 3 mg; vitamin B1, 1.8 mg; vitamin B2, 8 mg; vitamin B12, 0.01 mg; niacin, 30 mg; pantothenic acid, 10 mg; folic acid 0.55 mg, biotin, 0.15 mg. ^2^ ME was a calculated value, while the others were measured values.

**Table 3:** Effects of FMLP on growth performance of broilers.

Item ^1^	CON	3% MLP	3% FMLP	6% FMLP	9% FMLP	*p*-Value
1–28 d starter phase						
ADG (g/d)	27.4 ± 1.16 ^b^	30.6 ± 1.63 ^a^	30.3 ± 1.72 ^a^	29.8 ± 1.23 ^ab^	29.1 ± 1.90 ^ab^	<0.01
ADFI (g/d)	42.9 ± 2.17 ^b^	43.7 ± 1.91 ^ab^	46.1 ± 1.92 ^a^	42.1 ± 1.62 ^ab^	44.9 ± 1.82 ^ab^	0.0150
F/G	1.70 ± 0.140 ^a^	1.43 ± 0.120 ^b^	1.52 ± 0.100 ^b^	1.64 ± 0.120 ^ab^	1.54 ± 0.150 ^ab^	0.0140
29–56 d grower phase						
ADG (g/d)	34.4 ± 1.16 ^ab^	32.2 ± 1.57 ^b^	36.8 ± 1.89 ^a^	33.5 ± 1.96 ^ab^	31.0 ± 1.65 ^b^	0.0280
ADFI (g/d)	68.1 ± 3.87	70.4 ± 2.41	68.0 ± 1.99	71.5 ± 3.29	63.3 ± 4.20	0.383
F/G	2.05 ± 0.110	2.19 ± 0.100	1.92 ± 0.100	2.05 ± 0.180	2.20 ± 0.0700	0.442
1–56 d entire experimental phase						
ADG (g/d)	28.7 ± 1.92 ^b^	31.1 ± 1.67 ^ab^	34.0 ± 1.10 ^a^	31.5 ± 1.23 ^ab^	30.7 ± 1.25 ^ab^	0.0380
ADFI (g/d)	54.9 ± 2.73 ^b^	60.2 ± 5.83 ^a^	58.5 ± 3.35 ^ab^	60.1 ± 4.97 ^a^	56.9 ± 3.86 ^ab^	0.0280
F/G	1.85 ± 0.0400 ^ab^	1.93 ± 0.0800 ^a^	1.72 ± 0.0300 ^b^	1.91 ± 0.0800 ^ab^	1.89 ± 0.0300 ^ab^	0.0460

^a,b^ Values with different superscripts in the same row differ significantly (*p* < 0.05). ^1^ ADG: average daily gain; ADFI: average daily feed intake; F/G: feed to gain ratio. Con: control; 3% MLM, 3% FMLM, 6% FMLM, 9% FMLM, dietary supplementation of 3% mulberry leaf powder and 3, 6, 9% fermented mulberry leaf powder.

**Table 4:** Effects of FMLP on apparent nutrient digestibility of broilers.

Item ^1^	CON	3% MLP	3% FMLP	6% FMLP	9% FMLP	*p*-Value
Dry matter/%	79.6 ± 1.15 ^b^	82.1 ± 0.770 ^ab^	85.1 ± 0.860 ^a^	83.4 ± 0.810 ^ab^	84.0 ± 0.860 ^ab^	0.0290
CP/%	68.3 ± 1.65 ^b^	71.8 ± 2.22 ^ab^	75.4 ± 1.85 ^a^	72.8 ± 1.49 ^ab^	73.4 ± 1.57 ^ab^	0.0430
EE/%	76.3 ± 2.49	76.2 ± 2.72	77.5 ± 2.24	79.1 ± 2.91	80.0 ± 3.07	0.267
CF/%	28.9 ± 2.64 ^b^	26.3 ± 2.24 ^b^	30.7 ± 1.92 ^a^	30.1 ± 2.75 ^a^	31.8 ± 2.76 ^a^	0.0100
Ash/%	49.3 ± 3.66	48.8 ± 2.61	51.2 ± 4.82	48.8 ± 2.29	52.9 ± 3.29	0.349

^a,b^ Values with different superscripts in the same row differ significantly (*p* < 0.05). ^1^ CP: crude protein; EE: ether extract; CF: crude fiber. Con: control; 3% MLM, 3% FMLM, 6% FMLM, 9% FMLM, dietary supplementation of 3% mulberry leaf powder and 3, 6, 9% fermented mulberry leaf powder.

**Table 5:** Effects of FMLP on digestive enzyme activity in jejunum of broilers.

Item	CON	3% MLP	3% FMLP	6% FMLP	9% FMLP	*p*-Value
Amylase (U/mg prot)	466 ± 10.4 ^b^	557 ± 27.3 ^a^	538 ± 41.6 ^a^	530 ± 38.3 ^a^	522 ± 45.7 ^a^	<0.01
Lipase (U/mg prot)	59.4 ± 3.20 ^b^	73.3 ± 5.06 ^ab^	66.9 ± 8.28 ^ab^	69.4 ± 6.27 ^ab^	73.9 ± 5.93 ^a^	0.0210
Protease (U/mg prot)	246 ± 9.51	253 ± 9.36	260 ± 4.37	265 ± 5.9	274 ± 7.09	0.245

^a,b^ Values with different superscripts in the same row differ significantly (*p* < 0.05). Con: control; 3% MLM, 3% FMLM, 6% FMLM, 9% FMLM, dietary supplementation of 3% mulberry leaf powder and 3, 6, 9% fermented mulberry leaf powder.

**Table 6:** Effects of FMLP on intestinal tissue histomorphology of broilers.

Item ^1^	CON	3% MLP	3% FMLP	6% FMLP	9% FMLP	*p*-Value
Duodenum						
Villus height	962 ± 118	959 ± 116	1205 ± 187	1187 ± 113	1154 ± 131	0.216
Crypt depth	150 ± 12.0	137 ± 9.8	141 ± 14.7	148 ± 25.8	126 ± 10.618	0.423
V/C ratio	6.85 ± 1.58 ^c^	7.08 ± 1.30 ^bc^	8.61 ± 1.46 ^ab^	8.20 ± 1.72 ^ab^	9.16 ± 1.24 ^a^	0.0120
Jejunum						
Villus height	1074 ± 90.4	833 ± 46.5	974 ± 46.3	957 ± 78.8	741 ± 71.5	0.0500
Crypt depth	128 ± 13.3	146 ± 10.6	124 ± 12.5	153 ± 12.2	126 ± 13.9	0.519
V/C ratio	4.76 ± 0.760 ^b^	5.74 ± 0.810 ^b^	7.87 ± 0.510 ^a^	6.22 ± 0.580 ^b^	5.91 ± 0.420 ^b^	0.0260
Ileum						
Villus height	703 ± 69.7	753 ± 55.9	708 ± 57.9	667 ± 43.7	695 ± 35.0	0.553
Crypt depth	108 ± 15.7	98.4 ± 11.0	89.1 ± 14.3	91.7 ± 13.9	92.8 ± 16.7	0.128
V/C ratio	6.46 ± 0.520 ^b^	7.64 ± 0.270 ^a^	7.95 ± 0.640 ^a^	7.25 ± 0.960 ^ab^	7.54 ± 0.200 ^a^	0.0300

^a,b,c^ Values with different superscripts in the same row differ significantly (*p* < 0.05). Con: control; 3% MLM, 3% FMLM, 6% FMLM, 9% FMLM, dietary supplementation of 3% mulberry leaf powder and 3, 6, 9% fermented mulberry leaf powder. ^1^ V/C ratio: villus heigh to crypt depth ratio.

**Table 7:** Effects of FMLP on slaughter performance of broilers.

Item	CON	3% MLP	3% FMLP	6% FMLP	9% FMLP	*p*-Value
Dressing percentage, %	90.9 ± 0.449	88.4 ± 1.38	89.4 ± 0.630	89.8 ± 0.770	89.8 ± 0.680	0.375
Eviscerated carcass yield, %	70.5 ± 0.470	73.3 ± 0.930	68.0 ± 0.640	69.2 ± 0.600	69.4 ± 0.500	0.329
Breast muscle yield, %	15.8 ± 0.830 ^b^	15.9 ± 0.840 ^b^	17.2 ± 0.940 ^a^	15.7 ± 0.930 ^b^	15.8 ± 0.910 ^b^	0.0130
Thigh muscle yield, %	21.3 ± 1.02	20.3 ± 1.37	21.9 ± 0.680	21.2 ± 0.920	20.02 ± 1.05	0.874
Abdominal fat percentage, %	3.81 ± 0.860 ^a^	2.68 ± 1.05 ^b^	3.08 ± 0.690 ^ab^	3.00 ± 0.770 ^ab^	3.12 ± 0.640 ^ab^	0.0400

^a,b^ Values with different superscripts in the same row differ significantly (*p* < 0.05). Con: control; 3% MLM, 3% FMLM, 6% FMLM, 9% FMLM, dietary supplementation of 3% mulberry leaf powder and 3, 6, 9% fermented mulberry leaf powder.

**Table 8:** Effects of FMLP on breast and thigh meat quality of broilers.

Item ^1^	CON	3% MLP	3% FMLP	6% FMLP	9% FMLP	*p*-Value
Breast muscle						
Drip loss, %	3.48 ± 0.530	2.56 ± 0.460	2.56 ± 0.170	2.83 ± 0.360	2.61 ± 0.520	0.512
pH_45min_	5.87 ± 0.0700	6.06 ± 0.0900	6.13 ± 0.0400	6.10 ± 0.100	6.11 ± 0.0600	0.524
pH_24h_	5.72 ± 0.0300	5.75 ± 0.130	5.70 ± 0.0400	5.84 ± 0.0700	5.74 ± 0.0700	0.809
L*	50.2 ± 3.02 ^b^	53.8 ± 1.80 ^ab^	56.3 ± 2.32 ^ab^	54.4 ± 1.68 ^ab^	58.5 ± 1.74 ^a^	0.0430
b*	2.49 ± 0.440	2.63 ± 0.520	2.53 ± 0.430	2.39 ± 0.250	2.38 ± 0.260	0.991
a*	7.16 ± 0.620	8.02 ± 0.920	7.15 ± 0.980	7.31 ± 0.630	7.27 ± 0.670	0.973
Shear force, kg	1.44 ± 0.160 ^b^	1.69 ± 0.250 ^a^	1.53 ± 0.180 ^ab^	1.33 ± 0.170 ^b^	1.44 ± 0.390 ^b^	0.0120
Thigh muscle						
Drip loss, %	1.25 ± 0.370	1.24 ± 0.0200	1.17 ± 0.0100	1.14 ± 0.320	1.11 ± 0.0200	0.457
pH_45min_	5.98 ± 0.0600	6.45 ± 0.0900	6.46 ± 0.0600	6.49 ± 0.0500	6.25 ± 0.07	0.323
pH_24h_	5.60 ± 0.0500	5.77 ± 0.0800	5.84 ± 0.0500	5.71 ± 0.0400	5.73 ± 0.0700	0.685
L*	66.5 ± 2.18	56.9 ± 2.28	63.2 ± 3.01	61.3 ± 2.09	65.4 ± 2.41	0.851
b*	8.42 ± 0.130 ^a^	7.86 ± 0.320 ^ab^	6.87 ± 0.310 ^ab^	6.08 ± 0.630 ^b^	6.26 ± 0.510 ^b^	0.0320
a*	6.28 ± 1.54	5.27 ± 0.96	4.65 ± 1.05	5.09 ± 1.05	5.13 ± 1.55	0.733
Shear force, kg	1.40 ± 0.270 ^ab^	1.76 ± 0.760 ^a^	1.30 ± 0.410 ^ab^	1.25 ± 0.350 ^ab^	1.15 ± 0.520 ^b^	0.0150

^a,b^ Values with different superscripts in the same row differ significantly (*p* < 0.05). ^1^ L*: Lightness; a*: Redness; b*: Yellowness. Con: control; 3% MLM, 3% FMLM, 6% FMLM, 9% FMLM, dietary supplementation of 3% mulberry leaf powder and 3, 6, 9% fermented mulberry leaf powder. V/C ratio: villus heigh to crypt depth ratio.

**Table 9:** Effects of FMLP on chemical composition and IMP content in muscle of broilers.

Item ^1^	CON	3% MLP	3% FMLP	6% FMLP	9% FMLP	*p*-Value
Breast muscle						
Moisture %	74.3 ± 0.0700	73.3 ± 0.910	74.3 ± 0.650	74.2 ± 0.150	74.1 ± 0.630	0.186
EE %	1.40 ± 0.140	1.30 ± 0.170	1.38 ± 0.300	1.34 ± 0.160	1.39 ± 0.0600	0.345
CP %	22.1 ± 0.660	22.1 ± 0.510	22.0 ± 0.670	22.4 ± 0.250	22.3 ± 0.950	0.907
IMP mg/g	1.35 ± 0.0500 ^b^	1.52 ± 0.0600 ^ab^	1.77 ± 0.0100 ^a^	1.83 ± 0.0100 ^a^	1.79 ± 0.910 ^a^	0.0130
Thigh muscle						
Moisture %	75.8 ± 0.250	75.7 ± 0.440	75.9 ± 1.25	76.1 ± 0.720	75.2 ± 0.660	0.436
EE %	1.43 ± 0.110	1.35 ± 0.280	1.40 ± 0.210	1.47 ± 0.130	1.50 ± 0.240	0.958
CP %	19.7 ± 0.17	19.0 ± 0.810	19.14 ± 0.610	19.69 ± 0.730	20.25 ± 0.490	0.283
IMP mg/g	1.61 ± 0.0100 ^b^	1.78 ± 0.0300 ^b^	1.88 ± 0.0500 ^ab^	1.93 ± 0.0100 ^a^	2.01 ± 0.0400 ^a^	0.0390

^a,b^ Values with different superscripts in the same row differ significantly (*p* < 0.05). ^1^ CP: crude protein; EE: ether extract. IMP: Inosine monophosphate. Con: control; 3% MLM, 3% FMLM, 6% FMLM, 9% FMLM, dietary supplementation of 3% mulberry leaf powder and 3, 6, 9% fermented mulberry leaf powder.

**Table 10:** Effects of FMLP on amino acid profile in breast and thigh muscle of broilers.

Item ^1^	CON	3% MLP	3% FMLP	6% FMLP	9% FMLP	*p*-Value
Breast muscle						
TAA, %	17.9 ± 0.0500 ^b^	18.2 ± 0.0900 ^ab^	18.3 ± 0.0700 ^ab^	18.4 ± 0.0500 ^a^	18.5 ± 0.0600 ^a^	0.0190
EAA, %	8.23 ± 0.0600 ^b^	8.43 ± 0.0200 ^ab^	8.47 ± 0.0200 ^ab^	8.62 ± 0.0300 ^a^	8.78 ± 0.0800 ^a^	0.0300
DAA, %	6.53 ± 0.0500 ^b^	6.99 ± 0.0900 ^ab^	7.05 ± 0.0700 ^a^	7.09 ± 0.0900 ^a^	7.13 ± 0.0600 ^a^	0.0470
Thigh muscle						
TAA, %	18.2 ± 0.0100 ^b^	18.5 ± 0.0100 ^ab^	18.6 ± 0.0100 ^ab^	19.0 ± 0.0200 ^a^	19.0 ± 0.0100 ^a^	0.0260
EAA, %	8.63 ± 0.0100 ^b^	8.68 ± 0.0200 ^ab^	8.70 ± 0.0100 ^ab^	8.83 ± 0.0200 ^a^	8.88 ± 0.0200 ^a^	0.0340
DAA, %	7.23 ± 0.0100 ^b^	7.39 ± 0.0100 ^ab^	7.47 ± 0.0100 ^a^	7.62 ± 0.0100 ^a^	7.89 ± 0.0600 ^a^	0.0250

^a,b^ Values with different superscripts in the same row differ significantly (*p* < 0.05). ^1^ TAA: total amino acids; EAA: essential amino acids; DAA: delicious amino acids. Con: control; 3% MLM, 3% FMLM, 6% FMLM, 9% FMLM, dietary supplementation of 3% mulberry leaf powder and 3, 6, 9% fermented mulberry leaf powder.

**Table 11:** Effects of FMLP on fatty acid profile in breast and thigh muscle of broilers.

Item ^1^	CON	3% MLP	3% FMLP	6% FMLP	9% FMLP	*p*-Value
Breast muscle						
SFA	34.6 ± 1.72	34.9 ± 0.310	35.4 ± 1.24	35.2 ± 1.91	35.4 ± 0.840.	0.958
MUFA	25.6 ± 0.780	27.1 ± 0.860	28.9 ± 0.980	26.3 ± 0.290	28.3 ± 1.26	0.0750
PUFA	16.6 ± 0.800 ^b^	22.6 ± 2.30 ^a^	22.9 ± 2.07 ^a^	20.3 ± 2.49 ^a^	25.6 ± 3.04 ^a^	0.0420
EFA	9.84 ± 0.0600 ^c^	11.6 ± 0.160 ^ab^	11.8 ± 0.070 ^ab^	11.2 ± 0.200 ^b^	12.2 ± 0.0700 ^a^	0.0230
Thigh mucsle						
SFA	22.6 ± 1.66	24.5 ± 0.820	21.8 ± 1.73	21.7 ± 1.70	26.7 ± 2.80	0.132
MUFA	37.7 ± 5.63	38.9 ± 6.68	32.7 ± 2.17	38.4 ± 6.39	40.5 ± 6.05	0.652
PUFA	18.8 ± 2.70	19.5 ± 3.84	20.0 ± 2.23	20.3 ± 1.87	21.6 ± 1.05	0.232
EFA	13.7 ± 1.05	14.2 ± 1.39	15.5 ± 2.23	14.8 ± 1.59	15.8 ± 1.34	0.173

^a,b,c^ Values with different superscripts in the same row differ significantly (*p* < 0.05). ^1^ SFA: saturated fatty acids; MUFA: monounsaturated fatty acids; PUFA: polyunsaturated fatty acids; EFA: essential fatty acid. Con: control; 3% MLM, 3% FMLM, 6% FMLM, 9% FMLM, dietary supplementation of 3% mulberry leaf powder and 3, 6, 9% fermented mulberry leaf powder.

## Data Availability

The study did not report any data.

## Supplementary Materials

The following are available online at https://www.mdpi.com/article/10.3390/ani11113294/s1, Table S1: Effects of FMLP on amino acid profile in breast and thigh muscle of broilers. Table S2: Effects of FMLP on fatty acid profile in breast and thigh muscle of broilers.
