# Supplementary material for: Effects of Feeding Fermented Mulberry Leaf Powder on Growth Performance, Slaughter Performance, and Meat Quality in Chicken Broilers
Source: Animals (Basel). 2021 Nov 18;11(11):3294. doi: 10.3390/ani11113294 (PMC8614317; doi:10.3390/ani11113294)
Supplement: Supplementary file 1 [file animals-11-03294-s001.zip › animals-1448456-supplementary.pdf]

animals

Supplementary material

Effects of Feeding Fermented Mulberry Leaf Powder on Growth Performance, Slaughter Performance, and Meat Quality in Chicken Broilers

Yanan Ding [1], Xiaodie Jiang [1], Xiaofeng Yao [1], Haihan Zhang [1,2,3], Zehe Song [1,2,3], Xi He [1,2,3,*] and Rong Cao [1,2,3,*]

[1] College of Animal Science and Technology, Hunan Agricultural University, Changsha 410128, China; dingyanan13@163.com (Y.D.); jxd_0120@163.com (X.J.); yaoxiaofeng0525@163.com (X.Y.); zhhous@163.com (H.Z.); zehesong111@163.com (Z.S.)
[2] Hunan Engineering Research Center of Poultry Production Safety, Changsha, 410128, China
[3] Ministry of Education Engineering Research Center of Feed Safety and Efficient Use, Changsha 410128, China
* Correspondence: hexi111@126.com (X.H.); caorong19@126.com (R.C.)

Citation: Ding, Y.; Jiang, X.; Yao, X.; Zhang, H.; Song, Z.; He, X.; Cao, R. Effects of Feeding Fermented Mulberry Leaf Powder on Growth Performance, Slaughter Performance, and Meat Quality in Chicken Broilers. *Animals* **2021**, *11*, 3294. https://doi.org/10.3390/ani11113294

Academic Editor(s): Jarmo Valaja and Sylwester Świątkiewicz

Received: 19 October 2021
Accepted: 10 November 2021
Published: 18 November 2021

Publisher's Note: MDPI stays neutral with regard to jurisdictional claims in published maps and institutional affiliations.

Copyright: © 2021 by the authors. Licensee MDPI, Basel, Switzerland. This article is an open access article distributed under the terms and conditions of the Creative Commons Attribution (CC BY) license (http://creativecommons.org/licenses/by/4.0/).

Table S1. Effects of FMLP on amino acid profile in breast and thigh muscle of broilers.

Item[1]	CON	3%MLP	3%FMLP	6%FMLP	9%FMLP	*P*-value
Breast muscle						
Asp, %	1.65±0.0500[b]	1.66±0.0400[b]	1.80±0.0900[ab]	1.87±0.0800[ab]	1.98±0.0400[a]	0.0470
Glu, %	2.64±0.0100[b]	2.72±0.0500[ab]	2.76±0.2200[ab]	2.77±0.2900[ab]	2.82±0.0800[a]	0.0320
Ser, %	0.640±0.0100	0.590±0.0200	0.620±0.0100	0.630±0.0600	0.690±0.0900	0.857
His, %	0.790±0.0300	0.820±0.0800	0.850±0.0100	0.810±0.0700	0.860±0.0100	0.574
Gly, %	0.830±0.0900	0.790±0.0500	0.800±0.0500	0.820±0.1500	0.840±0.0300	0.458
Thr, %	1.08±0.0700	1.05±0.280	1.01±0.160	1.24±0.270	1.31±0.140	0.407
Arg, %	1.39±0.240	1.59±0.280	1.49±0.240	1.66±0.200	1.87±0.230	0.692
Ala, %	0.910±0.0700[b]	1.07±0.0600[ab]	1.18±0.0200[a]	1.15±0.0400[a]	1.19±0.0800[a]	0.0340
Tyr, %	0.630±0.0200	0.600±0.0100	0.580±0.0600	0.650±0.0700	0.610±0.0200	0.462
Val, %	1.13±0.0200[b]	1.22±0.0700[ab]	1.28±0.0100[a]	1.29±0.0300[a]	1.30±0.0800[a]	0.0320
Met, %	0.55±0.0400	0.52±0.0700	0.49±0.0100	0.59±0.0700	0.63±0.0200	0.0920
Phe, %	1.07±0.0400	1.05±0.0300	1.03±0.0500	1.12±0.0400	1.14±0.0300	0.578
Ile, %	1.03± 0.0200	1.00±0.0500	1.06±0.0200	1.10±0.0700	1.09±0.0600	0.186
Leu, %	1.63±0.0800	1.60±0.1100	1.61±0.0700	1.65±0.0900	1.61±0.0900	0.855
Lys, %	2.02±0.0300[b]	2.03±0.0500[b]	2.11±0.0100[a]	2.14±0.0700[a]	2.18±0.0300[a]	0.0410
Thigh muscle						
Asp, %	1.77±0.0100[b]	1.76±0.0200[b]	1.83±0.0100[ab]	1.89±0.0100[a]	1.94±0.0200[a]	0.0180
Glu, %	2.34±0.0100[b]	2.38±0.0100[ab]	2.36±0.0200[ab]	2.41±0.0900[ab]	2.62±0.0100[a]	0.0210
Ser, %	0.600±0.0100	0.620±0.0200	0.640±0.0700	0.660±0.0600	0.69±0.0900	0.464
His, %	0.760±0.0300[b]	0.850±0.0100[ab]	0.810±0.0100[ab]	0.890±0.0700[ab]	0.93±0.0100[a]	0.0450
Gly, %	0.800±0.0100	0.810±0.0500	0.850±0.01	0.820±0.15	0.870±0.130	0.255
Thr, %	1.00±0.0700	1.03±0.2800	1.11±0.360	1.12±0.270	1.15±0.140	0.307
Arg, %	1.42±0.240	1.45±0.390	1.48±0.260	1.69±0.200	1.77±0.230	0.491
Ala, %	0.960±0.0100[b]	1.07±0.0100[ab]	1.09±0.0200[ab]	1.15±0.0100[a]	1.20±0.0100[a]	0.0320
Tyr, %	0.610±0.0200	0.590±0.0200	0.640±0.0100	0.600±0.0700	0.620±0.0100	0.464
Val, %	1.23±0.0200[b]	1.32±0.0100[a]	1.38±0.0100[a]	1.37±0.0300[a]	1.40±0.0100[a]	0.0360
Met, %	0.490±0.0100[b]	0.50±0.0100[b]	0.53±0.0100[ab]	0.57±0.0100[ab]	0.64±0.0200[a]	0.0280
Phe, %	1.17±0.0100[ab]	1.05±0.0300[b]	1.16±0.0100[ab]	1.24±0.0100[a]	1.29±0.0100[a]	0.0460
Ile, %	1.09±0.0200	1.10±0.0100	1.13±0.0200	1.16±0.0200	1.17±0.0100	0.184
Leu, %	1.59±0.0100	1.56±0.170	1.64±0.0700	1.69±0.190	1.68±0.0900	0.356
Lys, %	2.22±0.0100[b]	2.26±0.0100[b]	2.19±0.0100[b]	2.32±0.0100[a]	2.39±0.0100[a]	0.0240

[a,b] Values with different superscripts in the same row differ significantly ($P < 0.05$). Con: control; 3%MLM, 3%FMLM, 6%FMLM, 9%FMLM, dietary supplementation of 3% mulberry leaf powder and 3, 6, 9% fermented mulberry leaf powder.

Table S2. Effects of FMLP on fatty acid profile in breast and thigh muscle of broilers.

Item[1]	CON	3%MLP	3%FMLP	6%FMLP	9%FMLP	*P*-value
Breast muscle						
C8:0	0.150±0.0100	0.140±0.0200	0.140±0.0100	0.145±0.0190	0.140±0.0200	0.671
C10:0	0.0200±0.0000	0.0200±0.0000	0.0200±0.0000	0.0200±0.0000	0.0200±0.0000	0.615
C12:0	0.0200±0.0000	0.0200±0.0000	0.0200±0.0000	0.0200±0.0000	0.0200±0.0000	0.816
C14:0	1.26±0.0600	1.45±0.160	1.28±0.0700	1.29±0.2000	1.29±0.0600	0.699
C16:0	21.5±1.73	21.8±2.65	21.3±2.90	21.1±2.32	21.8±3.53	0.930
C18:0	11.6±0.47	11.9±0.58	11.5±0.75	12.0±0.40	12.0±0.66	0.705
C20:0	0.0200±0.0000	0.02±0.0000	0.02±0.0000	0.02±0.0000	0.02±0.0000	0.805
C22:0	0.0200±0.0000	0.0400±0.0100	0.0300±0.0100	0.0300±0.0100	0.0400±0.0100	0.506
C16:1n-7	3.16±0.260	3.52±0.270	2.91±0.190	2.92±0.450	2.96±0.510	0.127
C18:1n-9t	22.4±1.48[c]	23.6±3.43[b]	23.7±5.17[b]	24.1±5.13[b]	25.2±5.15[a]	0.0430
C22:1n-9	0.0800±0.0000	0.110±0.0000	0.0800±0.0000	0.0900±0.0000	0.130±0.0000	0.215

C20:3n-3	5.94±0.0100	6.46±1.02	7.81±2.05	5.72±2.12	10.53±2.53	0.654
C20:5n-3	0.820±0.100	0.910±0.950	0.850±0.810	0.780±0.110	0.890±0.240	0.184
C22:5n-3	0.340±0.0800	0.330±0.160	0.420±0.0500	0.470±0.160	0.390±0.310	0.112
C22:6n-3	0.380±0.0100[b]	0.430±0.0500[a]	0.440±0.0200[a]	0.450±0.250[a]	0.470±0.0400[a]	<0.01
C18:2n-6	7.83±0.510[c]	9.03±1.43[b]	9.48±1.33[ab]	8.91±1.28[b]	9.77±1.22[a]	<0.01
C18:3n-6	0.590±0.0900	0.700±0.160	0.570±0.0600	0.560±0.0600	0.530±0.0100	0.382
C20:3n-6	0.820±0.1000	0.910±0.0400	0.850±0.0300	0.790±0.180	0.880±0.110	0.653
C20:4n-6	1.43±0.290[b]	1.87±0.430[a]	1.74±0.400[a]	1.73±0.390[a]	1.76±0.560[a]	<0.01
Thigh mucsle						
C8:0	0.270±0.0500	0.300±0.0200	0.320±0.0200	0.300±0.0340	0.370±0.0200	0.806
C10:0	0.0300±0.0000	0.0200±0.0100	0.0300±0.0010	0.0200±0.0100	0.0200±0.0000	0.728
C12:0	0.0700±0.0200	0.0800±0.0200	0.0600±0.0200	0.0700±0.0200	0.0900±0.0200	0.705
C14:0	0.560±0.120	0.590±0.0800	0.560±0.110	0.520±0.0500	0.590±0.0500	0.866
C16:0	21.90±1.63	23.8±1.87	21.2±1.82	21.0±1.67	23.7±2.74	0.103
C18:0	8.86±1.58	10.5±1.91	8.60±1.42	10.2±1.38	9.80±2.80	0.594
C20:0	0.0600±0.0100	0.0600±0.0100	0.100±0.0200	0.0600±0.0100	0.0800±0.0100	0.591
C22:0	0.0400±0.0100	0.0300±0.0100	0.0300±0.0100	0.0400±0.0100	0.0300±0.0100	0.972
C16:1n-7	5.90±0.250[b]	6.07±0.400[ab]	6.87±0.250[ab]	7.24±1.56[a]	7.02±0.560[a]	0.0360
C18:1n-9t	32.1±5.46	32.2±5.81	26.1±2.00	30.4±5.14	32.6±4.78	0.674
C22:1n-9	0.570±0.0200	0.620±0.140	0.560±0.0900	0.670±0.0700	0.770±0.0800	0.108
C20:3n-3	0.130±0.0100	0.0800±0.0000	0.0900±0.0100	0.120±0.0000	0.0700±0.0000	0.864
C20:5n-3	2.55±0.570	2.59±0.610	2.16±0.280	2.90±0.930	3.08±0.540	0.463
C22:5n-3	1.09±0.210	1.14±0.130	1.09±0.190	1.28±0.420	1.32±0.150	0.355
C22:6n-3	1.16±0.140	1.30±0.100	1.34±0.0600	1.71±0.740	1.68±0.220	0.305
C18:2n-6	13.3±2.20	13.7±2.31	14.9±2.11	14.2±2.54	15.1±2.59	0.221
C18:3n-6	0.360±0.0200[c]	0.390±0.0200[b]	0.430±0.0100[a]	0.44±0.0800[a]	0.44±0.0100[a]	0.0110
C20:3n-6	1.19±0.0600	1.35±0.230	1.12±0.130	1.16±0.200	1.21±0.150	0.475
C20:4n-6	1.35±0.100	1.36±0.0800	1.31±0.0800	1.47±0.260	1.48±0.0500	0.102

[a,b] Values with different superscripts in the same row differ significantly ($P < 0.05$). Con: control; 3%MLM, 3%FMLM, 6%FMLM, 9%FMLM, dietary supplementation of 3% mulberry leaf powder and 3, 6, 9% fermented mulberry leaf powder.
